# Impact of Bayesian Inference on the Selection of *Psidium guajava*

**DOI:** 10.1038/s41598-020-58850-6

**Published:** 2020-02-06

**Authors:** Flavia Alves da Silva, Alexandre Pio Viana, Caio Cezar Guedes Corrêa, Beatriz Murizini Carvalho, Carlos Misael Bezerra de Sousa, Bruno Dias Amaral, Moisés Ambrósio, Leonardo Siqueira Glória

**Affiliations:** 1 Laboratory of Plant Genetic Breeding (LMGV), Center for Agricultural Sciences and Technologies (CCTA), Universidade Estadual do Norte Fluminense Darcy Ribeiro (UENF), Av. Alberto Lamego 2000, 28013-602, Campos dos Goytacazes - RJ, Brazil; 2 Laboratory of Animal Science (LZO), Center for Agricultural Sciences and Technologies (CCTA), Universidade Estadual do Norte Fluminense Darcy Ribeiro (UENF), Av. Alberto Lamego 2000, 28013-602, Campos dos Goytacazes - RJ, Brazil

**Keywords:** Statistical methods, Plant breeding

## Abstract

Perennial breeding species demand substantial investment in various resources, mainly the required time to obtain adult and productive plants. Estimating several genetic parameters in these species, in a more confidence way, means saving resources when selecting a new genotype. A model using the Bayesian approach was compared with the frequentist methodology for selecting superior genotypes. A population of 17 families of full-siblings of guava tree was evaluated, and the yield, fruit mass, and pulp mass were measured. The Bayesian methodology suggest more accurate estimates of variance components, as well as better results to fit of model in a cross-validation. Proper priori for Bayesian model is very important to convergency of chains, mainly for small datasets. Even with poor *priori*, Bayesian was better than frequentist approach.

## Introduction

Perennial plant species such as guava trees (*Psidium guajava* L.) have specific characteristics such as a long reproductive cycle, a high annual variation in some traits as the yield, differences in precocity, and productive longevity^1^. This reduces the predictive power of the models, which most often means losses on invested resources. From the point of view of genetic improvement and use in commercial orchards, these characteristics have the following consequences: use of the same genetic material selected for an over number of years; the necessity of repeated evaluations in each individual throughout time, and the reduction in the survival rate of experiments during their useful life. The last one tends to generate unbalanced data that demand accuracy in selection methods^2^. So, using a method for modeling that produces more accurate results can undoubtedly save resources, and in the long time improve the chance of success of experiments with perennials plants.

Perennial plant breeding typically applies the procedure of Restricted Maximum Likelihood/Best Linear Unbiased Prediction (REML/BLUP) for the prediction of genetic values and estimation of variance components^2^. Mixed model theory has been a reference for assessing breeding programs in perennial plants, plants in general and animals^3^. Even though the frequentist methodology presents a number of useful properties, there is a limitation as the REML method only provides approximate confidence intervals^2^.

This can be avoided by Bayesian inference using an informative prior distribution with mixed models. This approach in genetic breeding, is founded on knowledge of *a posteriori* distribution. In this process, the likelihood function connects the *priori* (previous information of the experiment) to the posterior distribution, which finally contemplates the previous knowledge and the additional information obtained in the experiment.

Among the various Bayesian methodologies, the Markov Chains Monte Carlo simulation method can be applied for generate a chain of successive iterations updating the estimates by the likelihood starting from an initial parameter (*priori*). In the subsequent joint distribution the variances can be obtained, enabling the construction of more accurate confidence intervals (defined as probability intervals or credibility intervals), and also estimative of genetic parameters^4^.

The Bayesian approach have any advantages compared to the frequentist analysis. The main one is the possibility of using informative *priors* about parameters of the model^5^. In the frequentist’s approach, if you have previous data, you can even do a joint analysis with your current experiment, which is often hampered by the difference between outlines or even incomplete data. But this usually comes as a source of variation in the model and does not add much information beyond the possibility of identifying if the previous data are different from the current experiment. Another advantage is that the credibility intervals are close than the confidence intervals, if a proper *prior* has been used. Because the likelihood function, if a poor *priori* is used with mixed models, the performance of Bayesian with mixed models is at least equal to BLUP^6–8^.

This work aims to compare REML/BLUP and the Bayesian approach using a non-informative and a proper prior. For this, a superior performance of the Bayesian models is expected, observing the deviation of this methods in relation to phenotypic mean, for the selection of superior genotypes in a perennial population of *Psidium guajava*.

## Methods

### Genetic material and experimental design

A total of 17 families of full siblings was selected for this study, all of which belong to the Genetic Breeding Program of guava tree from the *Universidade Estadual do Norte Fluminense Darcy Ribeiro* (UENF), Rio de Janeiro, Brazil. Genotypes are derived from crosses between seven contrasting parents chosen by diversity genetics studies^9^. This population is in the final stages of the breeding program.

The experiment was performed in a randomized block design with two replicates. Each family was represented by 24 individuals (12 per block) with a total initially of 408 individuals. The experiment was conducted between 2016 and 2018. The spacing was of 3 per 1.5 m between rows and between plants, respectively. All culture treatments were applied according to the culture requirements^10^. Harvests were carried out at the individual level, where yield (kg.plant^−1^) was obtained, and generated one observation per individual because it’s a sum of production. For fruit mass (FM g) and pulp mass (PM g) were taken five observations in different fruits. Some genotypes were lost during the period of the experiments, which resulted in unbalanced data.

### Statistical model and analyses

First, we use the common methodology in the so-called frequentist breeding, and later we use the same model with the beyesian approach, using the mixed model:

1 $$y=Xb+Za+Wc+e$$

in which **y** is the observation vector; **b** is the parametric vector of the fixed effects (families), associated with the vector **y** by the incidence matrix known **X**; **a** and **c** are the parametric vectors of the random effects (block and individual within the family, respectively), also associated with **y** by the incidence matrices known, **Z** and **W**, respectively; and **e** is the residual vector, assuming that **a** and **c** ~ N (0, Gg e Ga) in which G is the genotypic and addictive variance matrix of the random effects and **e** ~ N (0, R) which R is the residual variance matrix of the random errors.

Was employed the method of restricted maximum likelihood (REML) to obtain the best estimates of variance components associated with non-orthogonal and unbalanced data^11^. The REML/BLUP method was executed using the *PROCMIXED* procedure in the *SAS* software^12^.

The Bayesian approach was used with the same model, applying the Monte Carlo method based on Markov Chains (MCMC), as described by Hadfield^13^, employing the *MCMCglmm::MCMglmm* package in *R* software^14^. A total of one million of iterations (*nitt*) were determined, discarding the first one hundred thousand first (*burn-in*) and performing a 1:3 (*thin*) sampling, totaling an chain with three hundred thousand iterations, where was obtained the variance components (*a posteriori* distribution). The Markov Chain convergence was tested by the Geweke criterion in accordance with the recommendations of Cowles and Carlin^15^ by using the *coda::geweke.diag* package^16^ in *R* software^14^.

The *a posteriori* means, credibility intervals, and standard deviation of the MCMC sample were obtained according to the generalized linear mixed model:

2 $${Y}_{lik}={\mu }_{i}+{b}_{ik}+{g}_{li}+{e}_{lik}$$

in which *Y*_*lik*_ is the *l-th* = [1,…,12] phenotypic value in the *i-th* = [1,…,17] family within the *k-th* = [1,2] block; *μ*_*i*_ is the overall mean of the *i-th* family; *b*_*ik*_ is the effect of the *i-th* family within the *k-th* block; *g*_*li*_ is the effect of the *l-th* individual within the *i-th* family; and *e*_*lik*_ is the residual term.

The joint data distribution (probability function) was utilized under the Bayesian approach: ${Y}_{ikl}|\beta ,g,{G}_{0},{R}_{0} \sim N({{x}^{\text{'}}}_{i}\beta +{{z}^{\text{'}}}_{ki}g,{\sigma }_{e}^{2})$, in which β is the vector of an *a priori* probability of systematic effects (overall mean); $g=\{{g}_{kl}\} \sim N(0,I\otimes {G}_{0})$ is the vector of an *a priori* probability of genotypic values, in which I is the identity matrix and *G*_0_ is the genotypic variance matrix; $e=\{{e}_{ikl}\} \sim N(0,I\otimes {R}_{0})$ is the vector of a prior probability of residual values with identical values of independent distribution, in which *R*_0_ with ${x}_{l}^{\text{'}}$ and ${z}_{l}^{\text{'}}$ are incidence vector relating systematization of the genotype effects for the corresponding phenotypic value; and ${\sigma }_{e}^{2}$ is the residual variance considered to be homogeneous. The prior information was based on meta-analysis or on the *posterior* distributions of the parameters from the previous cycle (2011–2015). The *priori* informative probability distribution for the fixed parameters of interest was obtained from provided by: ${\beta }_{i} \sim N({b}_{0},{V}_{b})$ in which *V*_*b*_ is a diagonal matrix of the *a priori* variance of *β*. An inverted Wishart distribution was adopted for each *G*_0_ and *R*_0_ as *a priori* for the covariance matrices: ${G}_{0} \sim {W}_{1}^{-1}({\Sigma }_{g},n)$ ande ${R}_{0} \sim {W}_{1}^{-1}({\Sigma }_{e},n)$, in which ${\Sigma }_{g}$ and ${\Sigma }_{e}$ are scale matrices.

The *posteriori* joint density of all the parameters, which are dependent on the genotypic effects of the respective matrix, but which assume *a priori* independence, is given by:

3 $$p(\beta ,g,{G}_{0},{R}_{0}|y)\propto p(y|\beta ,g,{G}_{0},{R}_{0})p(\beta |{b}_{0},{V}_{b})p(g|I\otimes {G}_{0})p({G}_{0}|{\Sigma }_{g},n)p({R}_{0}|{\Sigma }_{e},n)$$

A non-informative *priori* also tested in the model, using a standard *priori* of the function according with Hadfield^13^. This non-informative *priori* assumes for fixed effects a variance matrix ($V=I\times {1}^{10}$, in which *I* is an identity matrix) and mean equal to zero (mu = 0). Regarding the systematics effects, a variance equal to 1 (V = 1) and a parameter of degree of confidence around zero (nu = 0.002) were adopted. These distributions are equivalent to inverse gamma distributions (inverted Wishart).

A cross-validation scheme was tested in the methodologies. Ten folds were used in the cross-validation, in each fold the dataset was divided into two subsets, the fist was composed by 90% of dataset taken at random, and was used for training the model. The second (10% ~200 individuals) was the phenotypic values predicted by model obtained on the fist. In each fold a different subset was taken, until all the individuals that were evaluated had their predicted phenotypes.

## Results and Discussion

**Figure 1:**
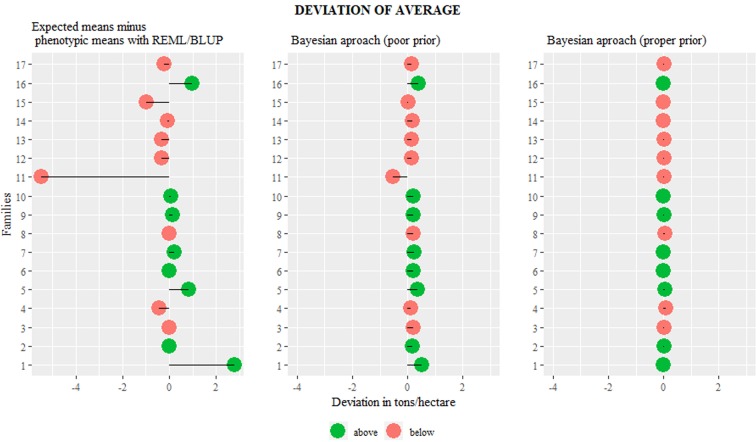
Differences between the mean estimates obtained by the REML/BLUP methodology and Bayesian inference and the phenotypic mean values in the total production variable (yield t.ha^−1^) in a full-siblings population of guava trees.

First, was applied the three methodologies throughout the data set, simulating one a common user, and we tried to observe some difference between the results obtained. Then, we plot the deviations of families mean and overall mean for the main yield trait (Fig. 1). Was possible to observe that the frequentist methodology presented a greater deviation, since in some cases the deviation reaches extreme values with errors of approximately 2.4 kg. It is worth mentioning that if this value is extrapolated to large areas of orchards, the difference can reach ~6 t.ha^−1^. In Bayesian approach with informative *priori*, it is noticed that the errors in relation to the average were constantly smaller.

As these estimates are part of the process in the mixed models applied to determine the variance components, to allow the addition of *prior* information improving the inference process. This analysis provides a more accurate description on the reliability of estimates and predictions than the REML method^17^, with much less simple methods^18^, even though the Bayesian inference has very similar goals to that of Fisher, in which the subjective element is removed from the choice of the *a priori* distribution.

After observing the deviations, was used a cross-validation to obtain model fit dispersion measures. It was considered as a good fit, the methodology that provided lower deviance information criterion (DIC) and also high values for a posterior adjustment probability of the model (Wprob) (Table 1). We verify the predictive power of the models through the correlation between the separate phenotypic data for validation and the prediction of the model obtained by training dataset, in each fold.

**Table 1:** Quality of fit models by cross-validation (10 folds: 90% training and 10% for validation), in the same sample sets of data for three methodologies: frequentist (REML/BLUP) and Bayesian (with prior no informative and prior informative) tested in the variables fruit mass (g), pulp mass (g) and yield (kg.plant^−1^) in *P. guajava*.

	Fruit mass			Pulp mass			Yield		
	DIC (SD/∆)	Wprob	r	DIC (SD/∆)	Wprob	r	DIC (SD/∆)	Wprob	r
A	14400.8 (94.5/221)	1.60E-25	0.66	18311.4 (1512.3/6049)	1.20E-71	0.31	7195.6 (110.1/322)	1.10E-228	0.70
B	14288.3 (56.1/184)	4.50E-01	0.76	17986.8 (1470.2/4752)	6.20E-01	0.36	6881.1 (238.6/709)	2.10E-160	0.76
C	14287.9 (56.0/183)	5.40E-01	0.81	17985.8 (1470.5/4752)	3.70E-01	0.37	6145.8 (81.4/257)	1.00E + 00	0.82

A = REML/BLUP; B = Bayesian without prior; C = Bayesian with prior; DIC = deviance information criterion; SD = standard deviation; ∆ (delta) = difference between the highest and lowest value of DIC; Wprob = model posterior probabilities; r = correlation between the Y predicted of model (training) and Y reserved for validation.

Bayesian with a *prior* showed the lowest DIC with 4287.9, 17985.8 and 6145.8 for the fruit mass, pulp mass and yield variables respectively, showing higher values of Wprob and correlation. With the standard deviations and the delta, it is possible to notice that among the folds of the cross validation, there was consistency in the fit of the model, with minor values for Bayesian inference with informative *priori*. Thus, whenever a random percentage of the data was used to test the model, it obtained very close results, mainly for the Bayesian approach than for the frequentist.

In the yield variable, where the setting with poor *priori* for Bayesian inference was worse than the frequentist. It was observed that a poor *prior* impaired the model as it can be observed in the DIC that although smaller than the frequentist had greater deviations between the folds of the cross-validation, result of the inconsistency of the model depending on the data. Since yield data consist of a single observation (total production), we can infer that Bayesian inference circumvents well the small dataset problem as long as an adequate *priori* is provided^19^.

**Figure 2:**
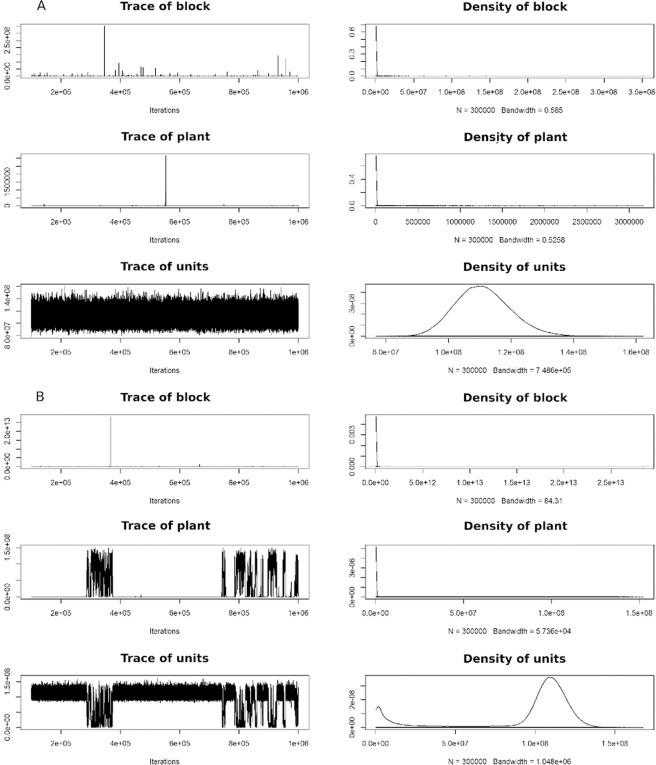
Distribution chain of mean estimates of 300k estimates for the variable yield in the sources of variation block, plants, and error (units) of the model using an informative *priori* (**A**) and a poor *priori* (**B**). On the right the density function of the distribution corresponding to the chain.

This accuracy arises because the MCMC method still exhibited great variations in the mean chains, therefore the lower significance, already justified by the greater consistency of the chain when starting from informative priori (Fig. 2). It is clear the great difference between the chains using an proper distribution for *priori* and a poor *priori*. Silva, *et al*.^20^ tested three distributions for informative *priori*, searching for the best model for variables in pigs. These authors also showed the difference in the accuracy that proper *priori* provides. The observation of the chain behavior is also a quality control criterion of the adjustment of the model to the data, given that the burn-in itself is a preventive measure to discard the inconsistent starting of the chain^21^. In this work, the importance of the informative *priori* is further evident when observing the chains of blocks, plants, and error (Fig. 2A,B).

It is also important to note that the stop iteration criterion in PROCMIXED is when the difference between the parameters of the distribution between one iteration and another is smaller than 1E-8^12^. In the Bayesian approach the chain of iterations is defined by the user (in this case 1 mi). At the onset of warming the MCMC method still produces estimates of averages with considerable variation, which tend to decrease with the increase in the chain^13^. When the user inserts a *priori* that represents the data well, providing good distribution parameters, that variation between one iteration and another is even smaller, and together with the excessive size of the chain, it generates more precise estimates^2^. We believe that the poor *prior* caused so much disturbance in the chain that not even the excessive size was able to stabilize the parameters and promote good distributions *posteriori* but it was still better than frequentist.

If was used a non-informative distribution for the parameters of the mixed model, Bayesian inference and BLUP should be equivalent. Thus a *priori* changes the *posterior* distribution, so that the information contained in it does not come only from the data (likelihood function)^6^. That is, it adds more information in the analysis, which is not based on the data. So, we proceeded with the selection of the individuals using Bayesian approach with proper *prior* to obtain the estimated means and predicted genotypic values. We believe to get more accurate genotypic values, because the Bayesian MCMC methods consider uncertainties in the parameters throughout the inference process. On the other hand the BLUP are predicted by point estimates of variance components and are used as true values, ignoring uncertainty in the variance parameters^22^.

**Figure 3:**
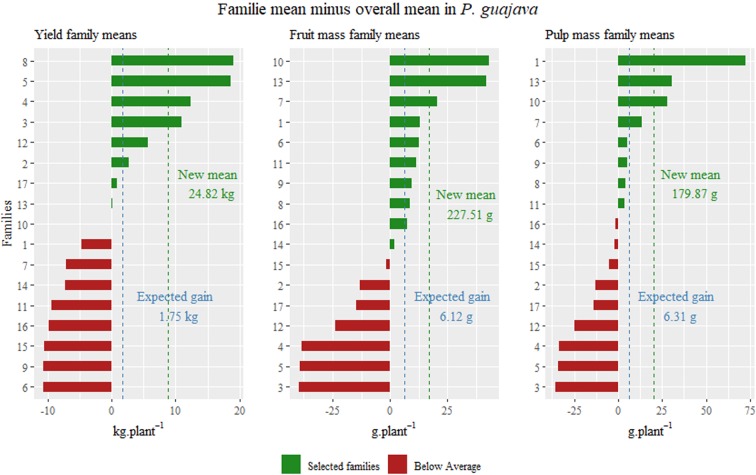
Estimated means in a population of full-sibs of guava trees obtained by Bayesian approach for yield, fruit mass and pulp mass traits.

The selection of the best families was performed to be recombined and to generate new populations. The objective is to increase the general population mean, and for this purpose the first nine families were selected, whose estimates were higher than the general average of the population (Fig. 3).

**Figure 4:**
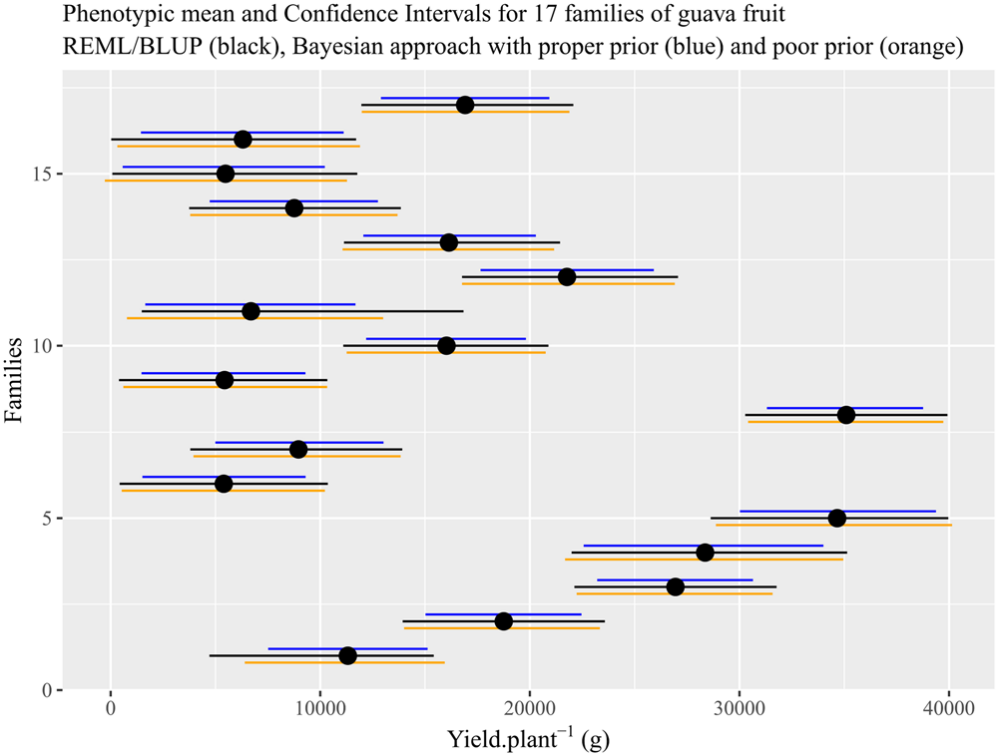
Phenotypic mean of the yield trait for the 17 families of Guava trees and the confidence intervals obtained by the REML/BLUP methodology and the credibility intervals obtained by the Bayesian approach with informative *priori* and poor *priori*.

The credibility intervals for this means were generally quite accurate, with a high degree of reliability. If we observe the credibility intervals for Bayesian and the confidence intervals for REML/BLUP, we can see better results with Bayesian inference (Fig. 4 and Table 2).

**Table 2:** Estimates of averages obtained through the frequentist methodology by REML/BLUP and by Bayesian inference (with poor *priori* and a proper *priori*) for the variables yield (kg.plant^−1^), fruit mass (g) and pulp mass (g) in *P. guajava*.

PROD				MF				MP			
F	REML/BLUP	BAYESIAN APROACH		F	REML/BLUP	BAYESIAN APROACH		F	REML/BLUP	BAYESIAN APROACH	
		poor prior	proper prior			poor prior	proper prior			poor prior	proper prior
8	35098,00***	34998,13***	35078,59***	10	271,80***	250,54**	250,73***	1	230,96***	231,91***	231,81***
5	34295,00***	34493,25***	34639,59***	13	248,71***	249,31**	249,24***	10	206,89***	190,29***	190,21***
4	28563,00***	28303,61**	28329,94***	7	228,47***	227,85**	228,04***	13	190,12***	187,76***	187,59***
3	26946,00***	26850,92**	26929,71***	1	222,38***	220,30**	220,37***	7	173,08***	173,07***	172,92***
12	21917,00***	21703,40**	21762,18***	6	220,34***	219,74**	219,86***	6	164,68***	164,83**	164,69***
2	18755,00***	18674,19**	18748,46***	9	217,96***	218,41**	218,86***	9	164,50***	164,68**	164,51***
17	17018,00***	16840,72**	16902,11***	11	217,13***	216,63**	216,83***	11	164,22***	163,99**	163,90***
13	16286,00***	16072,40**	16129,23***	16	216,34***	216,09**	216,16***	8	163,89***	163,69***	163,36***
10	15992,00***	15924,00**	16023,37***	8	216,19***	214,21**	214,82***	16	158,45***	158,40**	158,20***
1	10064,00***	11079,66**	11312,48***	14	209,23***	209,18**	209,18***	14	157,37***	157,58**	157,45***
11	9158,30**	8848,93*	8964,14***	15	204,26***	206,23**	205,82***	15	154,23***	154,40**	154,50***
7	8855,85***	8682,72*	8756,89***	2	194,24***	194,14**	194,24***	2	146,57***	146,71**	146,57***
14	8795,13***	6924,54*	6685,50*	17	192,38***	192,80**	192,71***	17	145,59***	145,91**	145,75***
15	5922,64*	6139,26*	6314,23*	12	182,88***	183,95**	183,75***	12	134,58***	134,79**	134,65***
16	5869,42*	5475,26 ^ns^	5480,41*	5	169,18***	169,33**	169,11***	4	125,90***	126,04**	126,12***
6	5395,73**	5339,30*	5428,43*	4	168,07***	167,81**	168,14***	5	125,67***	125,76**	125,51***
9	5368,02**	5305,03*	5394,13*	3	167,89***	167,79**	167,88***	3	123,63***	123,76**	123,65***
$\bar{X}$	16135,24	15979,73	16051,73		208,67	207,32	207,40		160,61	159,63	159,50

F = families (1,…, 17); ns = not significant; * = (*p-value* < 0,05); ** = (*p- value* < 0,01); *** = (*p- value* < 0,001) for the confidence intervals of averages. The first eight families were selected (from 13 upwards) of the table indicates the individuals that were selected with mean above the general average for yield trait, considering the Bayesian approach and proper *priori*. All values in the table are in grams (g).

This is because the REML method provides only approximate confidence intervals through the use of approximations and assumptions of asymptotic normality. The distribution and variance of the estimators are not known and, therefore questions regarding the effectiveness of the selection to be practiced cannot be answered with rigor. On the other hand, Bayesian analysis is based on the knowledge of the posterior distribution of the parameters, and allows the construction of exact confidence intervals (Bayesian probability intervals or credibility intervals)^17^.

Another part of the population was selected for test value of cultivation and use (VCU) (Table 3). These individuals were selected according to predicted genotypic values and gain estimates based on heritability (Table 4). Heritability estimates showed values within the expected range for the traits, considering that these are controlled by a large number of genes and are highly influenced by the environment^2^. The heritability also showed highs predict accuracy and standard deviation lowers. These measures are fundamental to planning the breeding program, allowing for more realistic forecasts of the next steps. Similar heritability were observed in guava fruit^10^, and even higher for this traits, but as shown in the standard deviation values were so high that they approached the estimates presented.

**Table 3:** Genotypic values and estimates of gains obtained through Bayesian inference for the variables yield (kg), fruit mass (g) and pulp mass (g) in *P. guajava*.

B	F	PL	Yield	EG (kg)	Fruit mass	EG (g)	Pulp mass	EG (g)
1	2	8	0.16	44.74	20.48	9.01	0.21	5.4
1	2	11	0.11	2.65	11.98	5.06	0.21	5.27
2	3	9	0.07	14.41	62.24	29.32	0.86	21.38
1	5	7	0.09	18.65	9.4	4.05	0.13	3.38
2	5	11	0.07	14.59	18.78	9.34	0.22	5.31
1	6	3	0.06	28.32	28.01	12.2	0.33	8.16
1	6	5	0.16	26.93	32.01	14.07	0.4	10.03
1	7	8	0.02	12.44	15.97	7.32	0.14	2.9
1	7	11	0.17	30.21	39.81	18.37	0.61	14.37
1	8	1	0.16	17.64	15.04	6.48	0.11	2.77
2	8	2	0.19	46.30	16.66	8.19	0.11	2.82
1	9	1	0.05	14.47	14.86	5.88	0.16	4.11
1	10	6	0.07	3.57	30.34	13.71	0.42	10.01
1	5	2	0.1	22.42	9.55	4.14	0	0.46
2	12	1	0.05	12.63	53.15	24.96	0.5	12.24
2	12	2	0.03	19.90	4.35	2.35	0.15	3.67
2	12	11	0.11	21.88	22.37	10.68	0.39	9.6
1	13	4	0.25	44.37	13.48	5.7	0.08	2.21
2	13	10	0.11	20.88	29.98	14.29	0.37	8.91
1	17	4	0.06	1.55	45.61	20.18	0.23	5.67
2	1	4	0.08	6.85	56.1	24.6	0.12	21.75
2	4	6	0	2.66	16.81	6.61	0.3	6.97
1	11	5	0.03	12.23	4.5	3.97	0.05	1.63
2	14	2	0.11	19	47.41	22.17	0.68	16.39
1	16	2	0.08	4.57	20.29	9.68	0.41	10.06
2	2	1	0.24	23.89	3.38	2.06	0.02	0.31
1	3	5	0.23	26.54	14.41	6.2	0.21	4.56
2	3	1	0.15	51.88	8.63	4.47	0.14	3.36
1	5	1	0.13	17.37	3.21	1.19	0.08	2.06
1	8	10	0.22	42.10	12.66	5.38	0.21	5.03

B = block; F = family of genotype; PL = id of genotype; EG = expected gain for individual mean based on each family mean and heritability.

**Table 4:** Heritability, predict accuracy and standard deviation values for the variables fruit mass (g), pulp mass (g) and yield (kg.plant^−1^) in *P. guajava* obtained with Bayesian inference.

	Fruit mass	Pulp mass	Yield
h^2^	0.36	0.31	0.20
Standard deviation	8.20E-03	7.35E-02	9.27E-03
Predict Accuracy	1.35	0.66	1.83
Overall mean	207.40 g	159.50 g	16.05 kg
Mean of selected	227.51 g	179.87 g	24.82 kg
Expected gain	6.12 g	6.31 g	1.75 kg

h^2^ = narrow-sense heritability.

Individuals were selected independently of the aim; industrial processes - where we consider the yield variable or in nature consumption - considering of greater interest the variables fruit mass and pulp mass looking for bigger and more vigorous fruits with less seeds and greater pulp mass. Since the components of variance were estimated through stochastic simulation (Gibbs sampling), we believe that the genetic values best represent the real value of the individual. The idea behind this argument is the exact analysis of finite-size samples because the data are fixed in the posterior distribution, instead of assuming multivariate normal distributions. Better statistical discussions on BLUP obtained by Bayesian inference may be found in^2,23–25^.

Perennial plant breeding programs have a particularity compared to annual plants. This difference is that the production period of perennials is very long. Therefore, the amount of resources needed to improve these species is much larger. Thus, to avoid estimation of variance components with less precision and thus make a program even more difficult, the Bayesian approach can be used. Another advantageous point of this approach is the possibility of using a *priori* information in the model. Thus, the breeder can make better use of the information available in the literature by using them as distribution measures in his model, instead of just comparing his results.

## Conclusions

In general, Bayesian inference provided the best fit of the model to this dataset, considering a population of full-siblings of *Psidium guajava*. This approach has provided a more complete and reliable result, thus allowing the selection of the best families to give continuity to the program and the best individuals to test crop value according to the expectations. The use of a *priori* information is the main advantage, and although it is subjective when the *prior* distribution is informative, the credibility intervals are narrower than the confidence intervals, and this is the main contributor to the accuracy of the model and help you bypass problems of small/unbalanced datasets.

Bayesian inference clearly has advantages over frequentist methodology, and with the advancement of computational powers this inference tends to become popular. We emphasize that we do not say that the Bayesian approach will be superior in all cases, but because of the advantages it can provide the investment to be tested it is worth it.

## Data Availability

The full phenotypic information, breeding values, scripts and chains generated used in this study, have been submitted at the *Open Science Framework* and was awarded the public doi identifier: 10.17605/OSF.IO/VKE6A.
